# Cost-effectiveness and economies of scale of a mass radio campaign to promote household life-saving practices in Burkina Faso

**DOI:** 10.1136/bmjgh-2018-000809

**Published:** 2018-07-16

**Authors:** Frida Kasteng, Joanna Murray, Simon Cousens, Sophie Sarrassat, Jennifer Steel, Nicolas Meda, Moctar Ouedraogo, Roy Head, Josephine Borghi

**Affiliations:** 1 Department of Global Health and Development, London School of Hygiene and Tropical Medicine, London, UK; 2 Development Media International CIC, London, UK; 3 Centre for Maternal Adolescent Reproductive and Child Health (MARCH), London School of Hygiene and Tropical Medicine, London, UK; 4 Centre MURAZ, Bobo-Dioulasso, Burkina Faso; 5 Africsanté, Bobo-Dioulasso, Burkina Faso

**Keywords:** health economics, health education and promotion, child health, cluster randomized trial

## Abstract

**Introduction:**

Child health promotion through mass media has not been rigorously evaluated for cost-effectiveness in low-income and middle-income countries. We assessed the cost-effectiveness of a mass radio campaign on health-seeking behaviours for child survival within a trial in Burkina Faso and at national scale.

**Methods:**

We collected provider cost data prospectively alongside a 35-month cluster randomised trial in rural Burkina Faso in 2012–2015. Out-of-pocket costs of care-seeking were estimated through a household survey. We modelled intervention effects on child survival based on increased care-seeking and estimated the intervention’s incremental cost-effectiveness ratio (ICER) in terms of the cost per disability-adjusted life year (DALY) averted versus current practice. Model uncertainty was gauged using one-way and probabilistic sensitivity analyses. We projected the ICER of national-scale implementation in five sub-Saharan countries with differing media structures. All costs are in 2015 USD.

**Results:**

The provider cost of the campaign was $7 749 128 ($9 146 101 including household costs). The campaign broadcast radio spots 74 480 times and 4610 2-hour shows through seven local radio stations, reaching approximately 2.4 million people including 620 000 direct beneficiaries (pregnant women and children under five). It resulted in an average estimated 24% increase in care-seeking for children under five and a 7% reduction in child mortality per year. The ICER was estimated at $94 ($111 including household costs (95% CI −38 to 320)). The projected provider cost per DALY averted of a national level campaign in Burkina Faso, Burundi, Malawi, Mozambique and Niger in 2018–2020, varied between $7 in Malawi to $27 in Burundi.

**Conclusion:**

This study suggests that mass-media campaigns can be very cost-effective in improving child survival in areas with high media penetration and can potentially benefit from considerable economies of scale.

**Trial registration number:**

NCT01517230; Results.

**Key questions**

**What is already known?**

- Few evaluations of the effectiveness of mass media interventions for child survival in low-income and middle-income countries have reported on cost-effectiveness, and none have reported with respect to mortality or disability-adjusted life years (DALYs).

**What are the new findings?**

- The cost per DALY averted of a 35-month mass radio campaign to prompt care-seeking for children with illness symptoms in rural Burkina Faso was $94 from a provider perspective and $111 from a societal perspective.
- The incremental cost-effectiveness ratio (ICER) fell below the national gross domestic product per capita threshold in almost all cases.
- When implementation at national scale in five African countries with varying media structures was modelled, the provider ICER varied between $7 and $27 per DALY averted.

**What do the new findings imply?**

- Mass media campaigns promoting behavioural change for improved child health can be a cost-effective complement to health system strengthening in low-income and middle-income countries with substantial potential for economies of scale.

## Introduction

Globally, child survival rates have increased markedly in recent years^1^; yet substantial inequities persist, within and across countries. Health system strengthening is essential to deliver effective interventions to improve child health in underserved and vulnerable populations. However, it is also important to tackle demand side barriers^2^ and increase community awareness of how to prevent illness and recognise danger signs for timely and appropriate care-seeking.^3^ Mass media is one approach to promote healthy behaviours to improve child health. With their considerable economies of scale, mass media campaigns have the potential to be highly cost-effective. However, this has not been widely assessed, particularly not in low-income and middle-income countries.^4 5^ To date, studies on the cost-effectiveness of mass media campaigns targeting child health promotion in sub-Saharan Africa have been limited in their ability to measure health outcomes.^6–8^ Existing evidence related to mass media campaigns in these settings address a single issue, most often reproductive health, nutrition, diarrhoea and immunisation, with radio as the most commonly used media,^5^ with no evidence on the cost-effectiveness of campaigns addressing multiple causes of child mortality.^9^

It is generally not feasible to conduct randomised controlled trials to evaluate the effect of mass media campaigns on health outcomes, given the difficulties of randomly controlling a campaign in national media, which often have the largest audiences. However, one such trial was recently completed in Burkina Faso^10^ where the strong presence of community radio stations and low national media penetration made a cluster randomised trial possible. This trial demonstrated that the media campaign increased health-seeking behaviours, specifically antenatal care attendance, health facility deliveries and primary care consultations for children under five. Further analysis showed that child consultation for the main diseases targeted by the campaign (malaria, diarrhoea and pneumonia) also increased significantly in the intervention zones relative to the control zones.^11^ We used a modelling approach based on evidence from the trial to estimate the incremental cost-effectiveness of a mass radio campaign promoting a range of important life-saving practices relating to illness in young children in Burkina Faso relative to a current practice scenario. We also projected the cost-effectiveness of a national scale-up in Burkina Faso and other countries in sub-Saharan Africa.

## Methods

### Study setting

In 2012, 1 in 10 children born in Burkina Faso died before the age of five. Malaria, preterm birth complications, pneumonia and diarrhoea were the main causes of mortality.^12^ Institutional delivery rates were 65% and despite reductions in maternal mortality, the maternal mortality ratio remained high (400 deaths per 100 000 live births).^13^ Seventy per cent of the population resided in rural areas with predominantly public healthcare provision.^14 15^ In 2002, user fees were removed for antenatal care in public facilities, and since 2006 childbirth and emergency obstetric care have been subsidised by the government.^16^ Child health services remained subject to user fees until mid-2016.^17^ Burkina Faso has a media environment dominated by local FM radio stations with a broadcasting range of 50–100 km and limited penetration of national media, which facilitated a cluster randomised controlled trial.^9^

### Intervention description

A mass radio campaign addressing the main causes of postneonatal child mortality was broadcast over a 35-month period between 2012 and 2014 by seven community radio stations in the country with high radio listenership.^10 18^ The campaign covered a population of approximately 2.4 million and comprised around 480 000 children under 5 years.^14^ The campaign consisted of 1-min radio spots, with a new spot each week broadcast 10 times every day, together with 2-hour long radio shows broadcast every weekday evening. Campaign messages addressed illness symptoms in children and the importance of taking the child to a health facility (or using oral rehydration salts for diarrhoea); nutrition during pregnancy and for neonates and infants; hygiene practices and antenatal care and institutional delivery (online supplementary appendix, p.1). Spots were translated into the six local languages spoken in the intervention areas. The spots and the radio show programme scripts were developed through formative research and piloting among people in rural villages and spots were recorded by a team of 17 radio professionals and actors based in Ouagadougou.^19^ The campaign was implemented by the Burkina Faso country office of the non-governmental organisation (NGO) Development Media International (DMI), with support from its international head office in London. Radio stations received mentoring and training, some equipment (laptops and software, solar panels) as well as a monthly cash payment to cover production costs of long format shows (payment to local actors, etc) equivalent to $1425 per station. In exchange, airtime (radio broadcasting time) was offered at no cost by the radio stations during the campaign. The intervention and theory of change are described in more detail elsewhere.^15^

10.1136/bmjgh-2018-000809.supp1 Supplementary file 1

### Costing methods

#### Approach to costing

We estimated the incremental financial and economic costs of the mass radio campaign compared with current practice (existing media activities) from a provider and a societal perspective. Costs incurred by the campaign implementers (provider costs) were measured during the start-up period (December 2010–February 2012) and throughout the campaign (March 2012–January 2015). Financial costs include the value of all financial transactions incurred as a result of the campaign. Economic costs value all resources at their opportunity cost, including donated resources. Research costs related to the evaluation were excluded as they did not contribute to the campaign impact. The additional costs to households from changes in care practices and care-seeking due to the intervention were quantified and added to provider (campaign) costs to measure societal costs. Facility-level costs relating to increased service uptake were not included, as primary care facilities were often underused, and drugs were paid for by patients.^20–22^ Although following the change to free child and maternal services in mid-2016 the costs of drugs would fall on the healthcare system, these costs are not included in the scale-up scenarios for 2018–2020 (see below) which are limited to a provider (campaign implementation) perspective.

#### Data sources

Provider costs were primarily estimated from financial accounts data. The quantities and value of resources not directly paid for were documented and valued at market prices. The value of airtime for spots and long format shows was estimated by radio stations. Household expenditure (including transport) for antenatal care, childbirth and care-seeking for children under five was estimated through a baseline survey of 5043 mothers of children aged under five in 2012.^18^

#### Analysis of costs

Provider costs were categorised by resource input: staff, travel, supplies, rent/utilities, equipment and vehicles and by activities. Start-up activities included the setup of operations, recruitment and training of staff, general administration, project coordination and contracting with radio stations. Recurring activities included general administration, project coordination, the development of radio spots and long format shows, regular support and mentoring of radio station staff and formative research (online supplementary appendix, p. 1). Time sheets were used to allocate staff time to activities and driver time sheets used to allocate vehicle costs. Capital items and start-up costs were annualised over the lifetime of the campaign.^23^

For economic costs, the 1 min spots were valued at market prices for bulk purchasing of advertising airtime. The long format shows were seen as providing a net contribution to radio stations due to the investment made by DMI in improving stations’ programming capacity, developing staff skills and were not costed in the base case analysis, but these costs were included in the sensitivity analysis.

Additional care-seeking costs to households due to the campaign were calculated from the mean costs of care-seeking for each service type reported in the baseline household survey. The care-seeking costs were then multiplied by the additional number of visits attributable to the intervention.

We present annual and total intervention costs over the 35 months of implementation. Provider costs were analysed in Excel and household costs were analysed in Stata. The cost per radio spot and long format show were estimated based on the share of staff time working on each, applying the same proportional allocation to divide the other activity costs between the two. All costs are presented in constant 2015 USD (exchange rates: 501 XOF/USD, 0.63 GBP/USD)^24^ (0.71 GBP/USD for the prospective 2018–2020 analyses) and were discounted at a 3% rate.

### Measurement of effectiveness

The effects of the mass radio campaign were measured on all-cause postneonatal under-five mortality (primary outcome) and under-five mortality (secondary outcome) in a cluster-randomised controlled trial.^10^ Fourteen community radio stations were selected for the evaluation. Clusters around each radio station were identified using the last national census with a population of about 40 000 inhabitants per cluster. We included villages located around the selected community radio station, with a good radio signal but more than 5 km away from town centres (thus less likely to be on the electricity grid, limiting access to television and making radio listening more likely). Seven clusters were randomly allocated to receive the intervention using pair-matched randomisation based on geography and radio listenership, as outlined further elsewhere.^10^ The trial was designed to detect a 20% reduction in the primary outcome (all-cause, postneonatal, under-five child mortality) with a power of 80% and had a 54% power to detect a 15% reduction at baseline. However, rapidly declining mortality in both arms over the study period (from 93.3 to 58.5 postneonatal under-five deaths per 1000 live births in the control group and from 125.1 to 85.1 in the intervention arm) further reduced the power of the study. The mortality reduction estimated from the endline survey showed no significant effect on mortality, but the CI was wide (risk ratio 1.00, 95% CI 0.82 to 1.22)^7^ and the lack of an effect on mortality appeared inconsistent with substantial increases in care-seeking observed in the intervention arm. This study therefore uses a modelling approach using the well validated Lives Saved Tool (LiST)^25^ to estimate the impact of the increase in health service utilisation observed during the trial period on the number of under-five lives saved and child mortality that may have been undetected by survey data.

### Analysis of health facility consultation data

Routine health facility data from across trial clusters were analysed using interrupted time-series analyses with mixed effects Poisson regression of monthly counts of attendances per cluster, to assess the intervention effect by time period on under-five consultations, for separate diagnosis categories over the period January 2011 to December 2014^10^. Findings showed that under-five consultations increased by 35% in year 1 (p<0.001), 20% in year 2 (p=0.003) and 16% in year 3 (p=0.049) in the intervention arm relative to the increase in the control arm; antenatal care consultations increased by 6% in year 1 (p=0.004), 9% in year 2 (p=0.026) and 8% in year 3 (p=0.129) relative to the increase in the control arm and facility-based deliveries increased by 7% in year 1 (p=0.004), 6% in year 2 (p=0.003) and 9% in year 3 (p<0.001).^10^

Further analysis of under-five consultations by diagnosis showed that consultations for malaria, pneumonia and diarrhoea, the three main diseases targeted by the media campaign (and the leading causes of postneonatal child mortality in Burkina Faso), also increased substantially in the intervention arm relative to the control arm.^11^ Consultations for malaria symptoms increased by 56% in the first year (p<0.001) of the campaign, 37% in the second year (p=0.003) and 35% in the third year (p=0.006); consultations for lower respiratory infections increased by 39% in the first year of the campaign (p<0.001), 25% in the second year (p=0.010) and 11% in the third year (p=0.525) and consultations for diarrhoea increased by 73% in the first year (p<0.001), 60% in the second year (p=0.010) and 107% in the third year (p<0.001). Consultations for other diagnoses which were not targeted by the radio campaign did not differ between intervention and control arms.^11^

### Modelling the effect of increased consultations on child mortality

As described in full elsewhere,^11^ we modelled lives saved due to augmented levels of care-seeking for maternal care and illness in children under five during the 3-year duration of the campaign using LiST.^25^ Briefly, increases in consultations for malaria, diarrhoea, pneumonia, antenatal care and facility deliveries were estimated using data from the trial clusters, as described above. For malaria, diarrhoea and pneumonia, we used the 2010 Demographic and Health Survey (DHS) data for rural populations in Burkina Faso to estimate the proportion of children taken to a health facility with symptoms of these conditions who received treatment. We then used these two sets of figures to estimate the increase in the proportion of children receiving appropriate treatment as result of the intervention. Baseline coverage levels (eg, the proportion of children taken to a health facility for symptoms of diarrhoea) were also estimated from DHS 2010. We took account of declining rates of under-five mortality by inputting mortality rates for each of the years of interest estimated from the trial endline survey. We used LiST to generate two sets of projections, one projecting mortality with the increased levels of consultations observed in the trial, the other with no changes other than in the underlying mortality rate. From these, we estimated that increased consultations should have resulted in 2967 under-five lives saved, representing an overall 7.1% reduction in child mortality (9.7% in year 1, 5.7% in year 2 and 5.5% in year 3).^11^

### Effectiveness measure

We estimated discounted disability-adjusted life years (DALYs) as discounted life years saved. Life years lived with disability were not included due to the lack of information on prevalence of long term serious sequelae from childhood illness or intervention impact on the duration of an illness episode in children. Life years saved per child were estimated based on the average life expectancy in Burkina Faso at age 2.5, 60 years, and discounted at 3% to 28 years^26^. This was multiplied by the number of lives saved to estimate total discounted life years saved.

### Cost-effectiveness analysis

In order to determine whether interventions are cost-effective, incremental cost-effectiveness ratios (ICERs) are estimated as the ratio of the difference in cost between the intervention and an alternative and the difference in effects between the intervention and an alternative. We estimated the ICER as the incremental cost per life saved and per DALY averted in children under 5 years of age relative to current practice from a provider (campaign) and societal perspective. This was done by combining the information on costs generated as outlined above, with information on programme effectiveness generated through the LiST model.

### Sensitivity analysis

A series of one-way sensitivity analyses were carried out to examine the effects of model and parameter uncertainty on the societal ICER, including variations in care-seeking behaviours and health outcomes, cost parameters and the discount rate (online supplementary appendix, p. 2). To assess the joint effect of uncertainty in model parameters, we ran a probabilistic sensitivity analysis (PSA) using 10 000 iterations, randomly sampling input parameters. Normal distributions were used for care-seeking behaviour and the lives saved estimates, and gamma distributions, bounded by 0 with a positive skew, were used for household costs.^27^ A mean point estimate was calculated by dividing mean costs by mean effects. The 95% of the ICER was based on a bootstrap of 1000 iterations of the PSA, also run with 1000 iterations.^28^ Its distribution is also presented graphically in terms of the 2.5th and 97.5th percentiles. We considered the ICERs in relation to three willingness to pay threshold values: those proposed in a 1993 World Bank report, updated to 2015 USD values as $41 (highly attractive intervention) and $248 (attractive intervention)^29^ and the WHO-CHOICE proposed country-specific threshold for a ‘highly cost-effective’ intervention of the national per capita purchasing power adjusted gross domestic product (GDP), $1700 in Burkina Faso in 2016^15^. We plotted the results as a cost-effectiveness acceptability curve.

### Scale-up scenarios

We conducted a number of scenario analyses, to project how costs would change according to the scale of implementation and with implementation in other contexts. The campaign was scaled up nationally in May 2015, covering an estimated 45% of the female population regularly listening to radio (versus an estimated 52% in the intervention area).^11^ As described in full elsewhere,^11^ the LiST model was used to estimate the impact of a nationwide campaign, using national level DHS data and assuming that a nationwide campaign would be 13% less effective as a result of lower radio listenership (ie, reflecting the percentage reduction in listenership from 52% to 45%).

We projected the incremental cost-effectiveness of the campaign at national scale in Burkina Faso during the time period of the trial 2012–2014, based on the actual costs incurred during the national scale-up in 2015, to explore what the ICER might have been had the intervention been implemented at national scale from the start. For household costs, our estimates of the mean cost per visit were applied to the projected number of additional visits in the national population.^14^

We also modelled the cost-effectiveness of ongoing implementation at scale during the period 2018–2020 in Burkina Faso and implementation at scale in four other African countries with diverse healthcare coverage, population size and media structure. The prospective scale-up scenarios included provider (campaign) costs only. Provider costs of scale-up in other African countries were estimated by adapting the incurred expenses in Burkina Faso to country-specific conditions in terms of salary levels, characteristics of the broadcasting market, production costs, costs of airtime and travel costs. We included the start-up costs of establishing a national campaign production office, as in Burkina Faso, with the number of full-time staff ranging from 14 to 35 per country. National offices were to be responsible for the development of radio spots, including formative research to inform content and monitoring of uptake, payments and support to radio stations. In countries where TV penetration was 20% or higher, we also included the costs of producing TV spots (online supplementary appendix, p. 3). Scale-up scenarios assumed less strategic and managerial input from the international NGO in London, with the London office costs constituting 9% of overall costs compared with 38% during the trial; valued spots based on airtime costs and excluded long format shows, as there was less evidence of impact on behaviours. Indeed, dose-response analysis at midline suggested a stronger correlation between behaviour change and spots exposure relative to longer format programmes exposure (regression coefficient, 0.9 vs 0.1).^18^ We assumed a 10% reduction in impact due to the exclusion of long-format shows from the campaign.

LiST was used to project mortality effects for 2018–2020 across the national populations of each country. The latest DHS data for each country were used to estimate the proportion of children with childhood illnesses (malaria, diarrhoea and pneumonia) who were taken to a health facility and received appropriate treatments and the increased care-seeking effects observed in the Burkina Faso trial, for each separate diagnosis category, were then applied accordingly. Coverage of antenatal care and facility deliveries were also estimated for each country, using the same approach as for the Burkina Faso projections. LiST was then used to project mortality effects for 3 year media campaigns in each country, from 2018 to 2020. Predicted campaign population exposure (penetration of used media channels) was based on national DHS estimates of radio and TV penetration in each country. We made the assumption that the number of people impacted was directly proportional to the number exposed. We therefore adjusted the mortality outcomes generated by the LiST modelling using the figure for female radio listening in Burkina Faso as a linear index.^10 11^

## Results

### Provider (campaign) costs

Economic start-up costs were $1 197 508 with administration and coordination activities consuming most of the costs. Annual economic costs of running the intervention over the study period amounted to $2 183 873 more than half of which were personnel costs. Material production constituted 28% of the running costs (17% for long format shows; 11% for spots); formative research and uptake monitoring an additional 8%. The support to radio stations was 13% of total running costs. The airtime value of spots amounted to 60% of the monthly cash payments to radio stations to support production costs (table 1 and online supplementary appendix, p. 4).

**Table 1:** Annual and total intervention costs by activity (2015 USD)

	Financial		Economic	
	Total cost over study period (%)	Annual costs, average over study period	Total cost over study period (%)	Annual costs, average over study period
Start-up costs	1 126 900 (15%)	375 633	1 197 508 (15%)	399 169
Running costs				
Project coordination	550 775 (7%)	183 592	550 775 (7%)	183 592
General administration	2 043 660 (27%)	681 220	2 048 515 (26%)	682 838
Formative research and uptake monitoring	594 570 (8%)	198 190	596 070 (8%)	198 690
Spot production cycle	818 744 (11%)	272 915	821 962 (11%)	273 987
Long format show production	1 331 004 (18%)	443 668	1 332 915 (17%)	444 305
Support to radio stations	981 955 (13%)	327 318	987 734 (13%)	329 245
Broadcasting (for spots, value of airtime)	0	0	213 649 (3%)	71 216
Total running costs	6 320 707 (85%)	2 106 902	6 551 620 (85%)	2 183 873
Total costs	7 447 608	2 482 536	7 749 128	2 583 043

### Household costs

Average household out-of-pocket expenditure in the intervention area was $2.40 for antenatal care, $3.25 for under-five consultations and $5.84 for childbirth (table 2). The intervention resulted in an estimated 423 787 additional visits. The estimated total household costs due to additional health facility visits linked to the intervention was $1 396 973.

**Table 2:** Household out-of-pocket costs in the intervention arm (direct medical costs and transport costs included) (2015 USD)

	Unit costs			Additional number of visits in the intervention area	Total cost
	Baseline survey sample	Out-of-pocket cost (% transport cost)		Incremental (intervention vs control) in 2012–2014 compared with baseline (2011)	Estimated incremental household costs in 2012–2014
	N	Mean	SD		
Antenatal care	2131	2.40 (9%)	5.98	29 619	71 003
Childbirth	1077	5.84 (18%)	8.56	17 189	100 322
Child under five illness consultation	346	3.25 (11%)	4.46	376 979	1 225 649
Total				423 787	1 396 973

### Intervention outputs and effectiveness

In total, the campaign broadcast 74 480 radio spots (152 individual spots) throughout the 35-month intervention period and 4610 long-format shows (based on 1317 long-format show modules (two modules per 2-hour show)). The total population size in the districts covered by the intervention was approximately 2.4 million at campaign initiation, including around 620 000 pregnant women and children under five who were considered to be direct programme beneficiaries. The intervention resulted in an estimated additional 423 787 contacts with health facilities (antenatal care, childbirth, children under five). This resulted in an estimated 2967 lives saved in children under five during the length of the campaign.

### Cost per output and cost-effectiveness

The provider cost per radio spot broadcast was $40 and $1040 per long format show broadcast. The annual provider cost per intended beneficiary was $12.5; the societal cost per beneficiary, which also included the additional cost of care-seeking for households, was $14.7. The provider (societal) cost per additional health facility visit was $18.3 ($21.6). The provider (societal) cost per life saved during the 3 years of the campaign was estimated at $2612 ($3083) and the provider (societal) cost per DALY averted $94 ($111) (table 3).

**Table 3:** Cost-effectiveness results (economic costs, 2015 USD)

Variables	Units	Provider cost*	Societal cost†
**Cost**			
Total cost of campaign		7 749 128	9 146 101
**Outputs**		**Provider cost per unit**	**Societal cost per unit**
Radio spots‡	74 480	40	n/a
Long format shows§	4610	1040	n/a
**Beneficiaries**		**Provider cost per person**	**Societal cost per person**
Population¶	2 378 990	3.3	3.8
Pregnant women and children under five^14^	621 254	12.5	14.7
**Behaviour change outcomes**		**Provider cost per unit**	**Societal cost per unit**
Additional number of facility visits (antenatal care, child births, under-five consultations)	423 787	18.3	21.6
**Health outcomes**		**ICER—provider**	**ICER—societal**
Lives saved in children under five (% mortality reduction)	2967 (–7.1%)	2612	3083
Life years gained	82 538	94	111
DALYs averted	82 538	94	111

*Radio campaign costs.

†Radio campaign costs+household costs.

‡1317 different modules each broadcasted once by seven radio stations.

§152 different spots each broadcasted 70 times each within a 1 week period by seven radio stations.

¶Annuaire Statistique 2011: Burkina Faso Ministere de la Sante, Secretariate General, Direction General de l’information et des Statistiques Sanitaires, 2012.

### Sensitivity analysis

**Figure 1:**
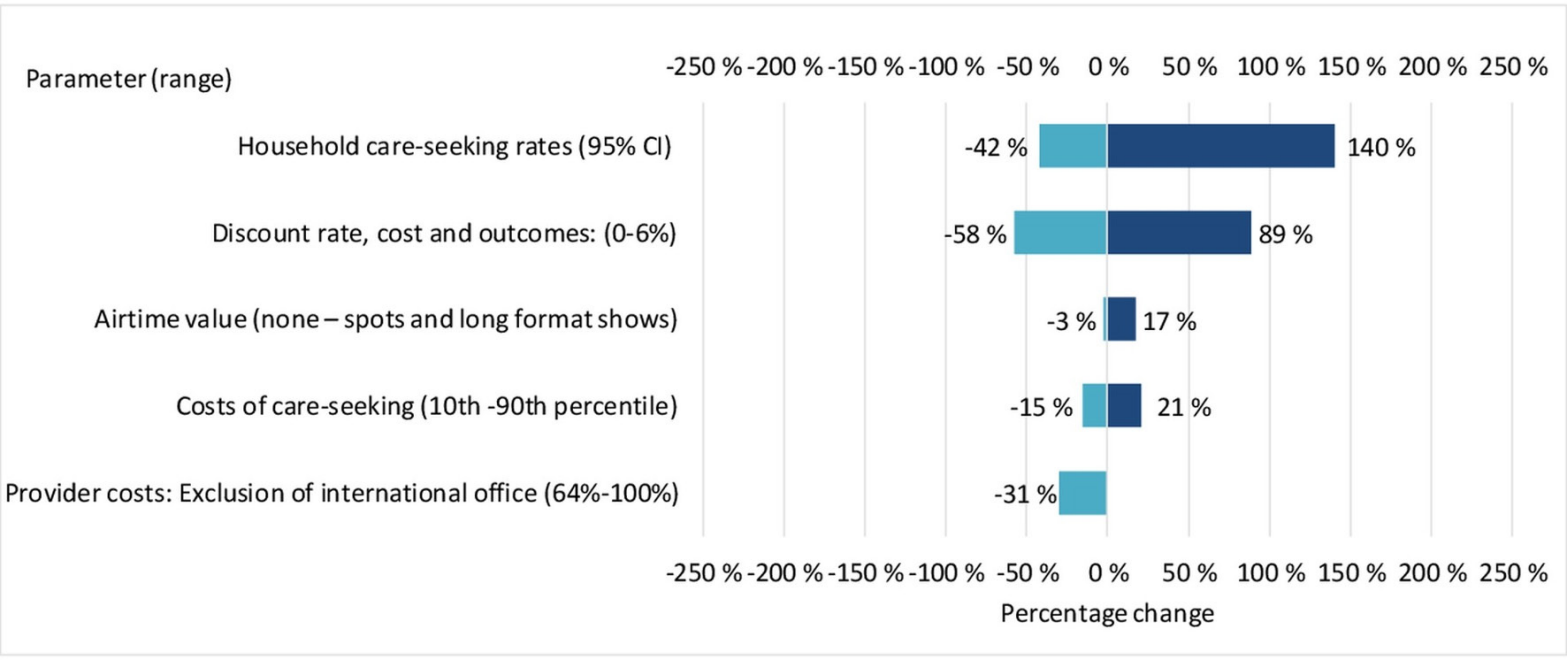
Tornado diagram of the percentage change in the base case ICER from one-way sensitivity analyses of key input variables. Light blue bars indicate that the input variable was at its minimum plausible value, whereas dark blue bars indicate that the input variable was at its maximum plausible value. The relative impact on the ICER is illustrated through the magnitude of the bars and the indicated percentages. ICER, incremental cost-effectiveness ratio.

The ICER was most sensitive to changes in the incremental number of facility consultations, which affected the modelled mortality effects and household costs, with the societal-perspective ICER varying between −42% and +140% per DALY averted (figure 1).

**Figure 2:**
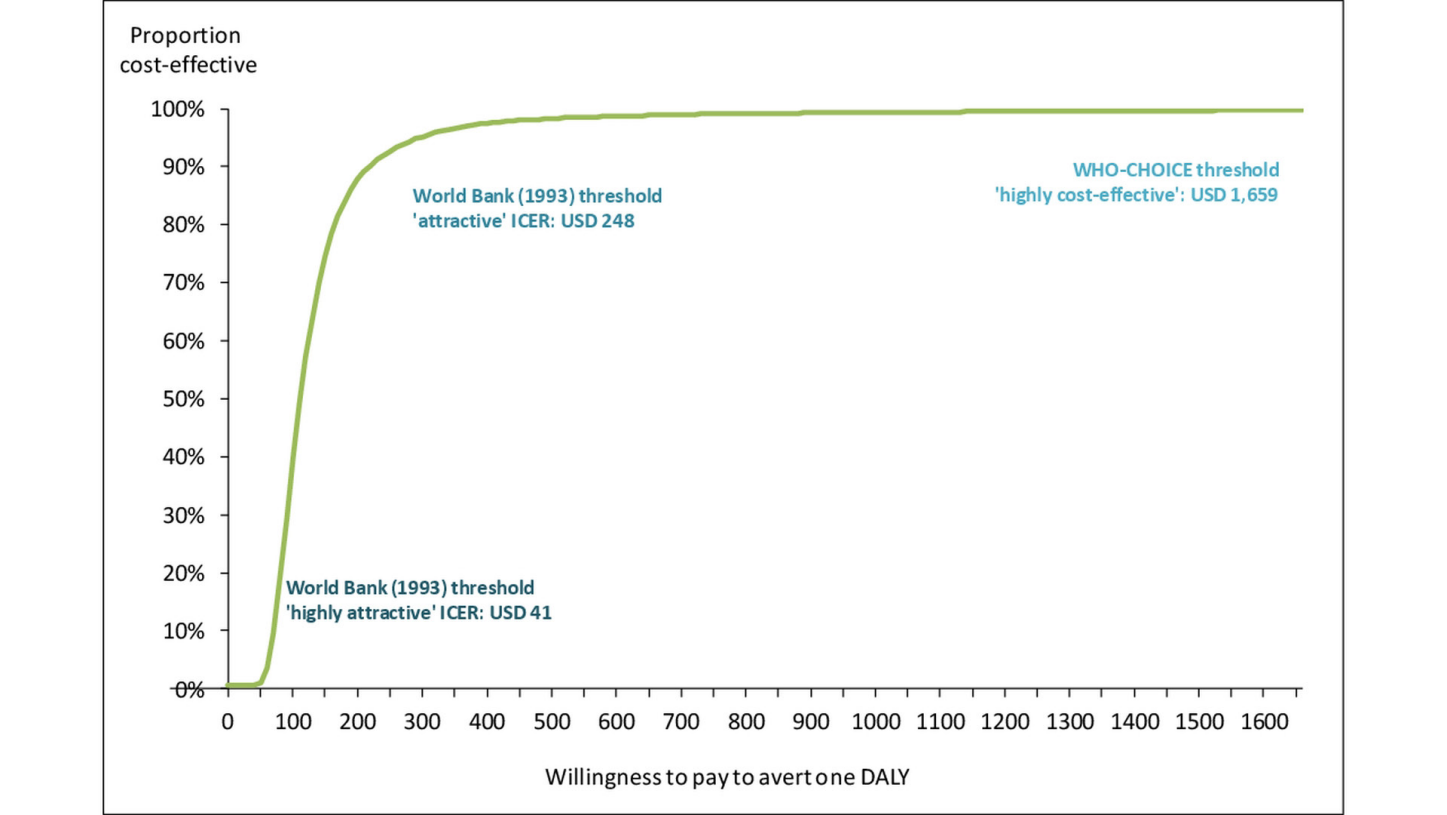
Acceptability curve. In our PSA, the within-trial analysis of the mass media campaign in Burkina Faso had a less than 1% probability of being ‘highly attractive’ but an 93% probability of being ‘attractive’ as per the generic low-income country thresholds suggested by the World Bank in 1993, updated to 2015 USD. It had a >99% probability of being ‘highly cost-effective’ in Burkina Faso based on the WHO-CHOICE suggested threshold of the country’s GDP per capita. DALY, disability-adjusted life year; GDP, gross domestic product; ICER, incremental cost-effectiveness ratio; PSA, probabilistic sensitivity analysis.

With a willingness to pay threshold of $248, the probability that the campaign was cost-effective is 93%, and at a threshold of the GDP per capita of $1700, it is >99% (figure 2). The 95% CI around the ICER resulting from the PSA was $−38 to 320 (around a mean of $153 due to the skewed costs data) (online supplementary appendix, p. 5).

### Scenario analysis

#### National scale-up

The projected total provider (societal) cost of a national campaign in Burkina Faso was $5 594 306 ($13 998 546) (table 4). The incremental provider (societal) cost-effectiveness of a national campaign in Burkina Faso during the trial period would have been $15 ($38) per DALY averted.

**Table 4:** Projected cost-effectiveness results of a nationwide 3-year campaign in Burkina Faso in 2012–2014 (economic costs, 2015 USD)

Variables	Units	Provider cost	Societal cost
**Cost**			
Total cost of campaign		5 594 306	13 998 546
Annual cost of campaign		1 864 769	4 666 182
**Beneficiaries**		**Provider cost per person**	**Societal cost per person**
Population^14^	16 248 558	0.3	0.9
Pregnant women and children under five^14^	4 258 740	1.3	3.3
**Health outcomes**		**ICER—provider**	**ICER—societal**
Estimated number of lives saved (range^11^)	13 400 (4349–27 389)	417 (204-1286)	1139 (204-1286)
Life years saved (range)	372 833 (121 004–762 054)	15 (7–46)	38 (18–116)
DALYs averted (range)	372 833 (121 044–762 054)	15 (7–46)	38 (18–116)

#### Forward looking scenarios

The projected ICERs for 2018–2020 varies between $7 and $27 in Burkina Faso, Burundi, Malawi, Mozambique and Niger, being lowest in Malawi and highest in Burundi (table 5).

**Table 5:** Cost-effectiveness scale-up scenarios 2018–2020 (2015 USD)

	Burkina Faso	Burundi	Niger	Malawi	Mozambique
Average annual provider (campaign) costs	1 811 584	1 623 943	1 890 430	1 176 473	2 747 424
Annual cost per population	0.10	0.14	0.09	0.07	0.10
Average annual number of lives saved in children under five	3010	2162	4069	5909	6433
Average annual number of DALYs averted	83 758	60 145	113 223	164 399	178 988
Cost per DALY averted	22	27	17	7	15

DALY, disability-adjusted life year.

## Discussion

We estimated the incremental cost-effectiveness of a mass radio campaign to increase child survival in rural Burkina Faso among a population of around 2.4 million. The cost per DALY averted was $94 from a provider perspective ($111 from a societal perspective). The intervention has potential for substantial economies of scale. Had it been implemented at national scale from the start, the cost per DALY averted would have reduced to an estimated $15 ($38) from a provider (societal) perspective. Cost-effectiveness is determined in relation to thresholds beneath which decision makers are willing to invest in interventions. In the trial setting, the ICER fell below the national GDP per capita threshold in almost all cases and had a 93% chance of being cost-effective in relation to the World Bank general threshold of ’attractive' health interventions in low income countries. However, the projected societal ICER for national scale implementation was just above the World Bank ’highly attractive' threshold of $41 (2015 USD). The campaign has the potential to be cost-effective in other African countries when implemented at national scale, with the provider ICER varying between $7 and $27 per DALY averted in selected countries. The estimated cost-effectiveness of the campaign varies between countries depending on the media landscape, penetration rates, population size and rates and causes of child mortality and was most cost-effective in settings with high media penetration rate. A mass media campaign for child survival will also be most cost-effective in settings with higher rates of child mortality.

The total estimated household costs of additional care-seeking from a national-scale programme in Burkina Faso exceeded the provider costs of the campaign. While the provider cost per DALY averted were considerably reduced due to the campaign’s economies of scale, the unit cost of care-seeking per household remains similar regardless of scale. Household costs were not included in the forward-looking scenarios. In Burkina Faso, the government took the decision to remove all user fees for child health services in 2016.^17^ Hence, in forward looking scenarios most of these costs—while remaining an economic cost component in the calculation—would fall on the government, with transport costs (approximately 10% of the total) remaining an out-of-pocket cost to households.

This is, to our knowledge, the first study to estimate the cost-effectiveness of a child health mass media campaign using final health outcomes or DALYs in a low-income setting.^5–8^ In comparison with other demand-side interventions for improved child health, the cost-effectiveness of the trial itself was similar to women’s groups (estimated ICERs of $43–411), hospital-promotion of breast feeding ($164)^30^ and nutrition/hygiene promotion programmes ($88–150) per life-year saved or DALY averted (all 2012 values).^31^ However, an important advantage of mass media campaigns is the potential for substantial economies of scale, since cost of media spots development and production remains the same regardless of number of broadcasts, and thus achievement of more favourable ICERs when operating nationally. For example, the ICER of women’s groups operating at a national scale was estimated to be 35% lower than that estimated in a trial setting^32^ whereas in our case in Burkina Faso, the ICER was estimated to reduce by 84% and 66% from a provider and societal perspective respectively for national scale compared with trial implementation. The projected ICER of a national-scale media campaign was similar to that of nutrition programmes for underweight children under five, facility-based integrated management of childhood illnesses and construction and promotion of basic sanitation for the prevention of diarrheal disease.^33^

There were a number of limitations to the study. Although we measured intervention effects through a cluster-randomised trial, the trial was unable to detect a mortality effect due in part to considerable falls in child mortality in both the intervention and control arms in the 3-year trial period. As there was evidence of the intervention increasing health facility utilisation,^10^ we modelled lives saved due to increased service use.^11^ The study found the intervention had different levels of effect on service use over time, and there is some uncertainty as to which is the appropriate measure of effect for a behaviour change intervention where effects are non-linear over time. In this case, we accounted for this year on year variation by taking the total estimated effect over the trial period to model outcomes. We do not know why the surge in under-five consultations in the first year of the campaign, particularly for malaria and pneumonia, fell in years 2 and 3 or why antenatal care or facility-based deliveries or diarrhoea consultations did not fall, and more generally how care-seeking behaviour is likely to change over time in response to campaign exposure. In sensitivity analyses, we modelled the uncertainty around care-seeking (using their 95% CI limit).^10 11^ There is also a question regarding the potential sustainability of behaviour change effects beyond the lifetime of mass media campaigns. Such effects were not included in our analysis, as experience in high-income countries suggest that a continuous effort, and therefore investment, is needed to maintain behaviour changes.^34 35^

The translation of service use into mortality gains is dependent on the quality of care delivered at health facilities, which was not observed within the study. While the modelling adjusted for the likelihood of children having received appropriate treatment, this was based on national estimates of treatment coverage from the 2010 DHS and may not accurately fully reflect quality at the time of the intervention in the intervention area. As a result, our estimate of intervention mortality effect may be biased. Due to lack of applicable uncertainty ranges, we did not model uncertainty around treatment quality (proportion of children whose symptoms required treatment, proportion who received appropriate treatments, the effectiveness of treatments and adherence to treatments) in the sensitivity analysis.

Our estimate of DALYs averted is potentially conservative for a variety of reasons. First, we restricted our estimate to DALYs saved in children as child health was the primary focus of the campaign. However, the observed increases in maternal healthcare utilisation resulting from the campaign may have resulted in additional DALYs averted due to maternal mortality reductions.^11^ Second, our DALY estimate does not include estimates of reductions in life-years lived with disability, due to the lack of information on prevalence of long-term serious sequelae from childhood illness or intervention impact on the duration of an illness episode in children. However, this effect would likely be minimal due to the relative short duration and infrequency of acute illness episodes.

The cost of the media campaign in Burkina Faso was based on actual incurred costs, with the exception of the valuation of airtime which was donated by stations; hence for this, estimated costs were used. There is some debate about the appropriate valuation of donated items. In financial costing, the cost was zero, but in economic costing, we used the price radio stations charge for commercial spots to value radio spots but did not include the airtime value of long-format shows due to their perceived value to radio stations in terms of building audiences and revenue. This issue ceased to be significant with the campaign design going forward at scale, where spot airtime was paid for and long-format shows excluded.

The ICER for the national scale-up in Burkina Faso and other sub-Saharan countries assumed that effects on care-seeking behaviour are the same as those observed in the Burkina trial. However, it may be difficult to achieve such effects outside of a trial setting. A number of country-specific contextual factors may also influence care-seeking behaviour and quality of care, yet due to lack of detailed country-level information, we could not provide valid uncertainty ranges around the cost-effectiveness estimates at scale.

## Conclusion

This study indicates that large-scale mass media campaigns encouraging prompt care-seeking for children with illness symptoms can be highly cost-effective in improving child survival.
