# Supplementary material for: Cost-effectiveness and economies of scale of a mass radio campaign to promote household life-saving practices in Burkina Faso
Source: BMJ Glob Health. 2018 Jul 16;3(4):e000809. doi: 10.1136/bmjgh-2018-000809 (PMC6058168; doi:10.1136/bmjgh-2018-000809)
Supplement: Supplementary file 1 [file bmjgh-2018-000809supp001.pdf]

Appendix

Table A Overview of project activities

Timing	Project activity	Description of inputs	Units of input
Start-up	Establishing the NGO	Fees to set up operations in Ouagadougou and London; staff time dedicated to operations set-up	3 FTEs* in Burkina Faso + 9 FTEs in London (including management, operations, finance, administration, programme development, creative director, research)
	Recruitment of management and administrative teams	Advertisements; staff time dedicated to recruitment	
	Project coordination	Management staff time	
	General operations	Office running costs	
	Recruitment and training of creative team	Advertisements; staff time dedicated to recruitment; staff time and other resources dedicated to training	Training of 13 FTEs in creative team - full-time training for the first two months
	Recruitment and training of formative research and uptake monitoring team	Advertisements; staff time dedicated to recruitment; staff time and other resources dedicated to training	Training of 3 FTEs in research team – one month
	Contracting with radio stations	Visiting stations & finding suitable partners, explaining campaign, negotiating prices	2 FTEs managing contracting process
Running	Project coordination	Management staff time and travel	36 FTEs in Burkina Faso + 8 FTEs in London (including management, operations, finance, administration, programme development, creative director, research)
	General operations	Office running costs	
	Formative research and uptake monitoring	Focus groups and in-depth individual interviews in rural villages to test media formats, monitoring of impact of spots/long format shows	In total around 75 research trips, visiting ~150 villages and interviewing a total of 4,024 people
	Spot production cycle	Writing, editing and initial selection, translation into pre-test languages. recording for pre-test, pre-testing, selecting final spots, translation into all languages, recording final spots in 6 languages	14 FTEs in creative team + 3 FTEs managers + 2 FTEs support staff
	Long format show production	Writing, editing and selection of final scripts. Scripts then sent out to radio stations where program teams practise and perform the scripts in local languages. DMI staff time spent training local stations to practise and perform scripts when visiting the radio stations	
	Support to radio stations	Capacity building and material support: training (one two-day training for radio station members), mentoring, equipment, CDs, financial support for running cost and contribution to solar power installation at one of the radio stations facing power outages that were affecting programmes	4 FTEs supporting the radio stations. Each time was visited approximately 40 times over the three years of the campaign
	Broadcasting	Value of airtime, for spots and long format shows (air time value of long format shows applied in sensitivity analysis): Both spots and shows were dominated by prompts for care-seeking in case of illness symptoms in children (spots 48%, shows 31%); followed by messages on nutrition - during pregnancy, of neonates and of infants (spots 22%, shows 20%); hygiene practices (spots 13%, shows 16%); and promotion of antenatal care visits and delivery at facility (spots 11%, shows 12%).	The 7 stations each broadcast 70 minutes per station per week for spots (one distinct spot per week) + 10 hours per station per week for long-format shows (10 different shows per week) throughout the 2.5-year period

**FTEs – full time equivalents*

Table B Parameters varied in sensitivity analyses

		One-way		PSA		
	**Parameter**	Base case (low, high)	Range interval	**Mean (SD)**	**Distribution**	**Source**
**Outcomes**	Under five mortality impact (modelled in LiST based on incremental health facility utilisation)	2,967(1,110-5,741)	95% CI of LiST model input values (facility consultations)	2,967 (1,181)	Gaussian	[1]
**Costs**	Incremental number of consultation children under five (linked to child mortality impact (above) and cost of care-seeking)	under fives: 376,979 (141,766-644,757)	95% CI	375,242 (140,217)	Gaussian	[2,3]
	Incremental number of consultation antenatal care (linked to child mortality impact (above) and cost of care-seeking)	antenatal care: 29,619 (1,120-60,446)	95% CI	29,520 (15,061)	Gaussian	
	Incremental number of facility childbirths (linked to child mortality impact (above) and cost of care-seeking)	childbirth: 17,189 (6,460-28,398)	95% CI	17,244 (5,599)	Gaussian	
	Household unit cost of care-seeking children under five (2015 USD)	under fives: 3.25 (0-7.63)	10th -90th percentile	3.25 (4.46)	Gamma	Baseline survey
	Household unit cost of care-seeking for antenatal care (2015 USD)	antenatal care: 2.40 (0-5.61)	10th -90th percentile	2.40 (5.98)	Gamma	
	Household unit cost of care-seeking for childbirth (2015 USD)	childbirth: 5.84 (0-13.47)	10th -90th percentile	5.84 (8.56)	Gamma	
	Provider costs (2015 USD)	7,749,128 (4,944,445-n/a)	Inclusion or not of international office costs: 100% (64%-n/a)	n/a	n/a	NGO accounts
	Airtime cost (2015 USD)	213,647 (0-1,831,277)	Spots only (No airtime value included – Spots and long format shows)	n/a	n/a	Campaign radio stations
**Discount rates**	Discount rate costs	3% (0-6%)	Standard practice in economic evaluation	n/a	n/a	[4]
	Discount rate LYS	3% (0-6%)	Standard practice in economic evaluation	n/a	n/a	

Table C Country indicators for scale-up analysis[5-7]

	Burkina Faso	Burundi	Niger	Malawi	Mozambique
**Population**					
Total population (million) 2015	18.1	11.2	19.9	17.2	28.0
Population residing in rural areas (%) 2015	70	88	81	84	68
**Media**					
Media structure	Localised	National	Mixed	Mixed	Mixed
TV penetration	Low	Medium	Low	Low	Medium
Radio penetration	Medium	High	Low	High	Medium
Cost of airtime	Low	Medium	Low	Medium	High
**Health**					
Fertility rate (births per woman) 2015	5.4	5.9	7.6	5.0	5.3
Maternal mortality rate (per 100,000 live births) 2015	371	712	553	634	489
Neonatal mortality rate (per 1000 live births) (2013)	27	29	27	22	27
Under-five mortality rate (per 1000 live births) (2013)	89	82	96	64	79
**Economic indicators**					
Total health care expenditure (THE) per capita (International $ PPP) 2014	82	58	54	93	79
Government expenditure (%) as a share of THE 2014	52%	53%	55%	53%	56%
Out-of-pocket expenditure (%) as a share of THE 2014	39%	21%	34%	11%	9%
GDP per capita (PPP Int. $) 2015	1,700	797	963	1,159	1,191

Table D Media adjustments made for each scale-up country scenario

Scale-up country	Media penetration (among women)	Data source	Adjustment applied to impact modelling*
Burkina Faso	45.2% weekly radio listening	DHS 2010	-13%
Burundi	57.9% weekly radio listening	DHS 2010	+12%
Niger	36.2% weekly radio listening	DHS 2012	-30%
Malawi	57.3% weekly radio listening	DHS 2010	+10%
Mozambique	60.0% weekly radio listening†	DHS2011	+15%

** The mortality outcomes generated by the LiST modelling was adjusted using the figure for female radio listening in Burkina Faso (52% as measured by the RCT endline survey) as a linear index.*

† There are huge discrepancies between the two most recent estimates for female radio listening in national surveys conducted in Mozambique: 42.5% in the 2011 DHS, 78% in the 2009 AIS. We have no reason to believe radio listening in Mozambique really did change that much between these two surveys and suspect this vast difference is a result of seasonal variation in the time the surveys were conducted. We have therefore take the midpoint of these two figures.

Table E Assumptions and methods in base-case and scale-up analyses

	Within-trial analysis 2012-2015	National scale-up scenario Burkina Faso	Prospective country scale-ups scenarios 2018-2020
**Costing perspective**	Societal	Societal	Provider
**Time period**	2012 March-2015 January	2012-2014	2018-2020
**Mass media format**	Radio spots and long format shows	Radio spots only (10% reduction of impact assumed without long format shows)	Radio (or TV) spots only (10% reduction of impact assumed without long format shows
**Media penetration**	52%	45%	45-60% (table C)
**Share of total cost, international (London) NGO office**	38%	9%	9%
**Number of staff running the programme (FTEs)**	44	14	14-35

Table F Start-up costs (Dec 2010 – Jan 2012) by activity (2015 USD)

	FINANCIAL	ECONOMIC
	**Start-up costs (%)**	**Start-up costs (%)**
Setting up the Burkina Faso office	15,651 (1%)	15,651 (1%)
Project coordination	175,004 (16%)	175,004 (15%)
General administration	604,337 (54%)	604,548 (54%)
Recruitment of management and administrative personnel	198,403 (18%)	198,403 (18%)
Recruitment of creative team	20,629 (2%)	20,629 (2%)
Recruitment and training of formative research and monitoring team	60,949 (5%)	60,949 (5%)
Contracting with radio stations	51,927 (5%)	51,927 (5%)
**Total start up costs**	**1,126,900**	**1,127,111***

**Differs slightly from the economic start-up costs in table 2 in the manuscript and in table F below since these are not annualised*

Table G Annualised running costs by input category (2015 USD)

	FINANCIAL		ECONOMIC	
	**Total cost over study period (%)**	**Annual costs, average over study period**	**Total cost over study period (%)**	**Annual costs, average over study period**
**Recurring costs**				
Personnel	4,287,060 (58%)	1,429,020	4,287,060 (56%)	1,429,020
Travel	726,692 (10%)	242,231	726,692 (9%)	242,231
Supplies (incl communication costs)	767,371 (10%)	255,790	767,371 (10%)	255,790
Rent and utilities	329,839 (4%)	109,946	329,839 (4%)	109,946
Air time	0	0	213,649 (3%)	71,216
**Total recurring costs**	**6,110,963 (82%)**	**2,036,988**	**6,324,612 (82%)**	**2,108,204**
**Capital costs**				
Radio station equipment	59,989 (1%)	19,996	63,626 (1%)	21,209
NGO office/media production equipment	147,541 (2%)	55,846	114,068 (1%)	59,231
Vehicles	42,208 (1%)	14,069	49,314 (1%)	16,438
**Total capital costs**	**209,745 (3%)**	**69,915**	**227,008 (2%)**	**75,669**
**TOTAL RUNNING COSTS**	**6,320,707 (85%)**	**2,106,902**	**6,478,322 (84%)**	**2,183,873**
Start-up costs	1,126,900 (15%)	375,633	1,197,508 (16%)	399,169
**TOTAL COSTS**	**7,447,608**	**2,482,536**	**7,749,128**	**2,583,043**

Figure A Cost-effectiveness plane showing the statistical uncertainty around cost and DALYs in the cluster-randomised trial

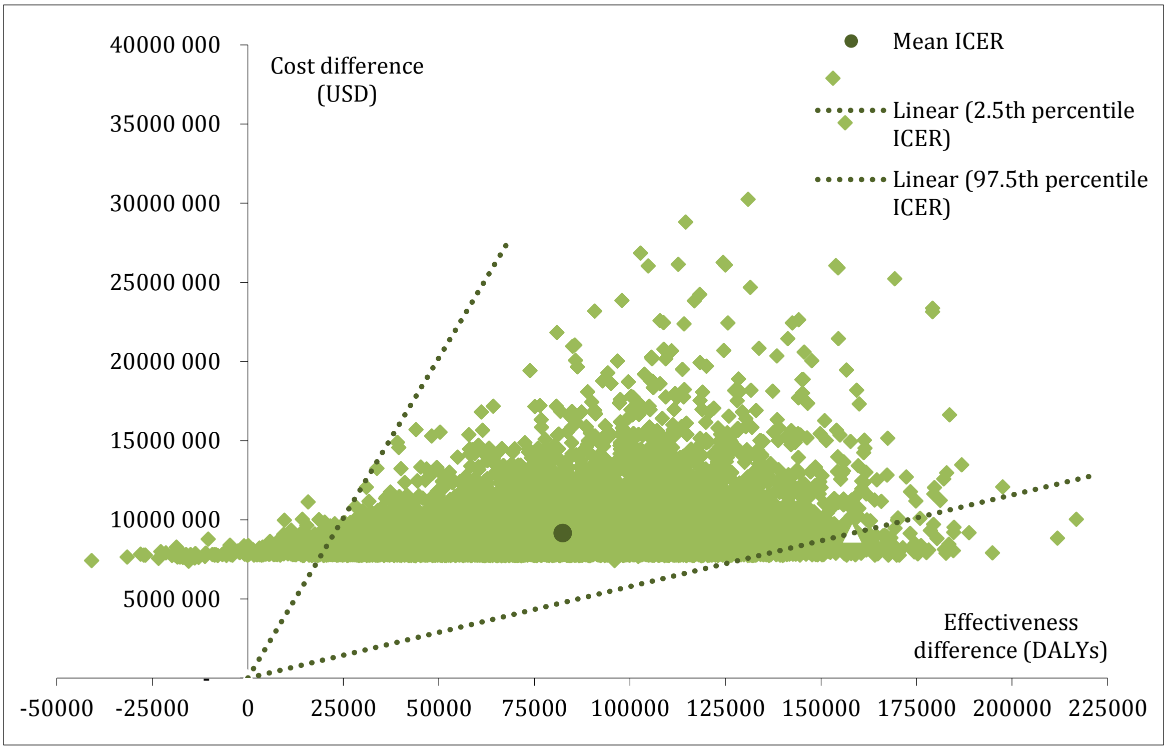

The plotted results of the probabilistic sensitivity analysis illustrate its distribution in a cost-effectiveness plan, with 10,000 dots each representing the results of one of the simulations. The scatter-plot is cut off at the level of the provider cost of the campaign - as these were the measured costs occurred they were not varied in the within-trial analysis. The larger plot represents the median ICER in the simulation, USD 111. The 2.5th and 97.5th percentiles of the ICER are pictured as dashed lines. Ninety-five percent confidence intervals were calculated through a bootstrap with 1,000 times 1,000 iterations resulting in a 95% CI of $-38-320 around the mean value of the PSA ($153).

References

1. Murray J, Head R, Remes P, et al. Estimating the effect of a mass radio campaign on child mortality in Burkina Faso using the Lives Saved Tool (LiST): modelling from a repeated cross sectional cluster randomised trial. *[In review]* 2017.
2. Annuaire Statistique 2011: Burkina Faso Ministere de la Sante, Secretariate General, Direction General de l'information et des Statistiques Sanitaires, 2012.
3. Sarrassat S, Meda N, Badolo H, et al. Effect of a mass radio campaign on family behaviours and child survival in Burkina Faso: a repeated cross-sectional, cluster-randomised trial. *Lancet Glob Health* 2018; **6**(3): e330-e41.
4. Tan-Torres Edejer T, Baltussen R, Adan T, et al. WHO Guide to Cost-effectiveness Analysis. Geneva, Switzerland, 2003.
5. UNICEF. The State of The World's Children 2016 Country Statistical Information. 2016.
6. WHO. Global Health Observatory. 2016.
7. World_Bank. World Bank Open Data. 2016.
